# Transcriptome Analysis Revealed Potential Regulatory Networks Underlying Corolla Movement in *Mirabilis jalapa* (Nyctaginaceae)

**DOI:** 10.3390/biology15070585

**Published:** 2026-04-06

**Authors:** Dingkun Liu, Huiqi Yan, Xuan Wang, Xiaohong Yan, Bing Zhou

**Affiliations:** Key Laboratory of Jiangxi Province for Biological Invasion and Biosecurity, School of Life Sciences, Jinggangshan University, Ji’an 343009, China

**Keywords:** corolla movement, *Mirabilis jalapa*, molecular mechanism, transcriptome analysis

## Abstract

Corolla movement is a typical plant movement behavior that enables plants to optimize pollination and adapt to environmental changes. Nevertheless, its molecular mechanism remains poorly understood. In the present study, we conduct a comprehensive transcriptome analysis of *Mirabilis jalapa* (Nyctaginaceae) corolla at five stages (AG-EG) to elucidate the regulatory networks underlying movement. The results showed that the differentially expressed genes (DEGs) were mainly associated with cellular processes, catalytic activity, MAPK signaling, plant hormone signal transduction, and photosynthesis-related pathways, highlighting their involvement in corolla dynamics. Transcriptome profiling further demonstrated that auxin, ethylene, and abscisic acid signaling pathways were key hormonal regulators of corolla movement. Moreover, Ca^2+^ transport genes (CNGCs and CMLs) and respiratory burst oxidase homologs (RBOHs) were significantly enriched, indicating that Ca^2+^–ROS signaling oscillations also play an important role in driving differential cell expansion and turgor changes. Transcription factor analysis also revealed the upregulation of *WRKY2*, *WRKY22*, and *WRKY33*, suggesting that WRKYs act as the critical transcriptional regulators linking ROS–Ca^2+^ signals with downstream gene expression. The reliability of RNA-Seq data was confirmed by RT-qPCR, which showed high consistency with transcriptome profiles. These findings suggested that corolla movement in *M. jalapa* is carried through the integration of hormonal pathways, Ca^2+^–ROS signaling, and WRKY-mediated transcriptional regulation. This research provided novel insights into the molecular basis of plant movement and established a foundation for further study on floral dynamics and adaptive strategies in angiosperms.

## Simple Summary

Corolla movement is a common adaptive behavior in flowering plants, and it helps plants improve pollination efficiency and adapt to changing environments, yet the molecular mechanisms controlling this process are still poorly understood. This dynamic floral trait is directly related to plant reproductive success, and exploring its regulatory basis can provide valuable clues about how plants adapt to their surroundings. In this study, we conducted a comprehensive transcriptome analysis of *Mirabilis jalapa* (Nyctaginaceae) corollas at five distinct movement stages to uncover the underlying regulatory networks. We found that genes with changed expression levels were mainly involved in basic cellular processes, catalytic reactions, and key pathways related to cell signaling, hormone regulation, and photosynthesis. We identified auxin, ethylene, and abscisic acid—key plant hormones that regulate growth—as core regulators of corolla movement. Notably, genes related to calcium transport and reactive oxygen species (ROS) production were highly enriched, indicating that calcium–ROS signaling drives the cell expansion and pressure changes that power corolla movement. We also found that WRKY family transcription factors, which control gene activity, were upregulated and likely act as key links between upstream signals and downstream responses. The reliability of our data was verified by RT-qPCR, and these findings fill a major knowledge gap, offering new insights into the molecular basis of plant movement and supporting future research on plant adaptive evolution.

## 1. Introduction

Plants are immobile throughout their life cycle; nevertheless, they possess tissues, organs, and individual cells capable of perceiving environmental cues. These responsive movements are very crucial to optimize their survival, growth, and reproduction. For instance, the *Venus flytrap* (*Dionaea muscipula*) rapidly folds its leaves upon stimulation of its sensory hairs, and the guided elongation of a pollen tube as it delivers sperm cells to the ovule. These intriguing phenomena have fascinated biologists since Darwin’s seminal work “*The Power of Movement in Plants*” [1]. Over the past century, researchers have extensively studied plant movements, which are defined as changes in the spatial orientation or configuration of organs or their parts [2]. Plant movements are generally categorized into three types (tropic, nastic, and autonomous) based on the stimulus and driving force [2,3]. Tropic movements, such as irreversible differential growth of stems during phototropism, are induced by directional signals, such as light and gravity. Nastic movements, such as reversible turgor changes that cause leaf folding, are triggered by non-directional stimuli, such as temperature and touch. Autonomous movements are driven by endogenous biological clocks, resulting in circadian behaviors such as the rhythmic opening and closing of leaves, flowers, and stomata [2,3].

Among these movement forms, flower opening is quite remarkable because it exposes the reproductive organs to promote cross-pollination, which is essential for the reproduction of most angiosperms [4]. This process is primarily driven by corolla (petal) movement, which occurs through several mechanisms such as differential growth between the adaxial and abaxial petal surfaces in response to temperature, humidity, water status, circadian regulation, and hormones [4,5]. In *Lilium* (Liliaceae), petal opening mainly results from edge growth [6], while in *Ipomoea* (Convolvulaceae), the opening and closing of the corolla depend on differential cell expansion across the midrib rather than the lamina [7]. Despite these extensive studies, the molecular mechanisms underlying corolla movement remain incompletely understood. Recent work in *Rosa hybrida* (Rosaceae) revealed that asymmetric cell expansion at the petal base, controlled by an *RhPMP1–RhAPC3b* regulatory module in an ethylene-dependent manner, drives petal movement [8].

Most plant species display epinastic petal movement and bend downward toward the pedicel during flower opening [2]. However, certain taxa, such as *Mirabilis* (Nyctaginaceae), exhibit more complex and specialized corolla movements. The mature flowers of *Mirabilis* are zygomorphic, with a trumpet-shaped corolla formed by five fused petals [9,10,11,12]. During opening, the corolla also undergoes epinastic bending similar to other species, but its final posture provides additional adaptive advantages for pollination. The open corolla acts as a visual cue to attract and guide pollinators [13,14], forms a stable landing platform, and shields nectar and reproductive organs to prevent desiccation and prolong pollinator visits [15]. It also functions as a biomechanical filter that restricts access to only strong pollinators capable of pressing the corolla to reach the reproductive structures [16,17]. These specialized roles illustrate that corolla movement in *Mirabilis* is not only a mechanical process but also an evolutionary adaptation that enhances pollination efficiency and reproductive success.

The phenomenon of flower closure is relatively common in Gentianaceae plants, with a series of related studies conducted on groups such as *Gentiana algida* and *G. straminea* [18]. These studies mainly focused on corolla movement and flowering phenology. However, the molecular mechanisms regulating this phenomenon have not yet been reported. Research on the corolla movement of *M. jalapa* is still in the stage of morphological description and ecological function studies. For instance, Yu et al. [11] discovered that temperature significantly affects the corolla movement of *M. jalapa*. However, they only analyzed the internal and external factors of corolla movement from the perspective of morphological anatomy and physiology. Chen et al. [19] discussed the position of the stigma in the corolla during the flower closure phenomenon of *M. jalapa* and its significance in adaptive evolution. Hu et al. [10] treated the corolla of *M. jalapa* to investigate the impact of the corolla on the reproductive fitness of the plant. Therefore, based on previous research results, this study explored the molecular mechanisms underlying the corolla movement phenomenon of *M. jalapa* based on transcriptome analysis.

**Figure 1:**
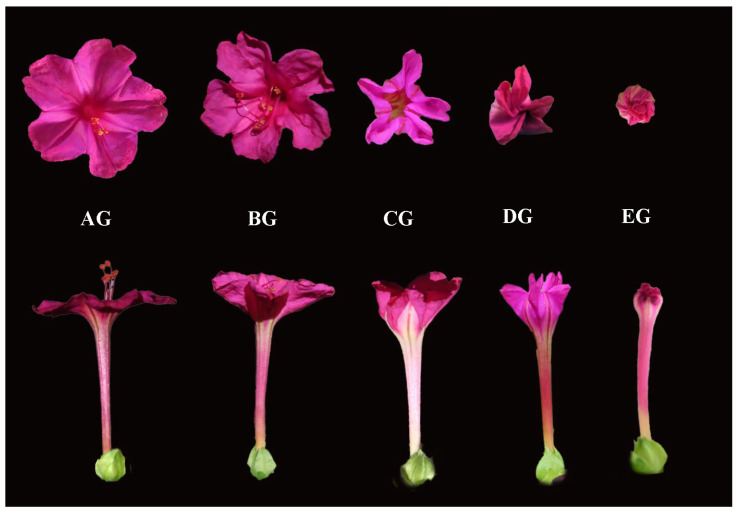
Morphological stages of corolla closure in *Mirabilis jalapa*. Five sequential stages were defined based on visible morphological changes during corolla closure: fully expanded corolla stage (AG), early closure stage (BG), intermediate closure stage (CG), late closure stage (DG), and completely closed corolla stage (EG).

Corolla movement in flowering plants is a dynamic process involving coordinated cellular expansion, turgor regulation, and signal transduction. In Mirabilis jalapa, the corolla exhibits characteristic movements during anthesis, including opening followed by gradual closure accompanied by bending and twisting of the floral tube. However, the molecular mechanisms underlying this process remain largely unknown. In this study, we hypothesized that corolla closure may be associated with coordinated regulation of plant hormone signaling, Ca^2+^ signaling, reactive oxygen species (ROS), and transcriptional regulators that influence differential cell expansion. To explore this possibility, transcriptome sequencing was performed across five sequential stages of corolla closure: the fully expanded corolla stage (AG), early closure stage (BG), intermediate closure stage (CG), late closure stage (DG), and completely closed corolla stage (EG) (Figure 1). Comparative transcriptome analysis was conducted to identify candidate genes and regulatory pathways potentially involved in corolla movement.

## 2. Materials and Methods

### 2.1. Plant Material

*M. jalapa* plants were propagated from seeds and cultivated in 10 × 10 cm pots filled with a substrate mixture of nutrient soil and vermiculite (3:1, *v*/*v*). The plants were maintained in a controlled greenhouse environment at 24 °C under a 16 h light/8 h dark photoperiod and a light intensity of approximately 2000 lx. After 30 days of growth, uniformly developed and healthy plants were selected for sampling. To investigate corolla movement, the corolla morphology of *M. jalapa* was observed and recorded at 3 h intervals from the initial opening stage (AG) to the complete closure stage (EG) using a digital camera, and the corolla opening angle was measured with a protractor to quantify the movement process. For comparative transcriptomic analysis, corolla tissues were collected at five distinct stages (AG–EG) corresponding to the observed corolla movement stages. The sampling workflow included quick dissection of corolla tissues to avoid RNA degradation, followed by immediate processing. For each stage, three independent biological replicates were obtained. All samples were immediately frozen in liquid nitrogen and stored at −80 °C until RNA extraction and subsequent physiological analyses [20].

### 2.2. RNA Extraction, Library Preparation, and Sequencing

Total RNA was isolated from corolla tissues using the TRIzol reagent (Invitrogen, Carlsbad, CA, USA) following the manufacturer’s instructions [21]. RNA purity and concentration were measured using a NanoDrop spectrophotometer (Thermo Fisher Scientific, Waltham, MA, USA). Moreover, RNA integrity was assessed with an Agilent 2100 Bioanalyzer (Agilent Technologies, Santa Clara, CA, USA) [22]. Samples with RNA integrity number (RIN) values greater than 7.0 were used for library construction. cDNA libraries were prepared with the NEBNext^®^ Ultra™ RNA Library Prep Kit (New England Biolabs, Ipswich, MA, USA) [23] and sequenced on the DNBSEQ platform at the Beijing Genomics Institute (BGI, Shenzhen, China) [24]. Raw reads were processed to remove adapter sequences, low-quality reads, and reads containing excessive ambiguous bases, yielding high-quality clean data [25]. The clean reads were aligned with the *M. jalapa* reference transcriptome, and gene expression levels were quantified as fragments per kilobase of transcript per million mapped reads (FPKMs) [26]. The raw sequencing data assembled results have been deposited in figshare, and the data are publicly available at https://doi.org/10.6084/m9.figshare.31898767 (accessed on 2 April 2026).

### 2.3. Assembly and Annotation

*De novo* transcriptome assembly was conducted using Trinity (version 2.11.0) [27]. The quality of assembled unigenes and transcripts was evaluated based on GC content, mean length, and N50/N90 statistics [28]. Functional annotation of the assembled sequences was performed using BLASTX (version 2.11.0) searches (E-value < 1 × 10^−5^) against multiple public databases, including NR, Swiss-Prot, KEGG, GO, and Pfam [29,30].

### 2.4. Differential Gene Expression and Enrichment Analysis

RNA-seq data quality was validated by PCA based on rlog-normalized expression values and by calculating Pearson correlation coefficients between biological replicates, with sample clustering heatmaps generated to confirm high reproducibility across replicates. Differentially expressed genes (DEGs) among the five corolla stages (AG–EG) were identified using the DESeq2 package (version 1.38.0) [31] with the criteria |log_2_FoldChange| ≥ 1 and adjusted *p*-value ≤ 0.01. GO enrichment analysis was performed using the GO::TermFinder package (version 0.86) [32], while KEGG pathway enrichment was conducted in R 4.2.2 using the phyper function [33]. GO terms and KEGG pathways with Q-values ≤ 0.01 were considered significantly enriched. Visualization of DEG expression patterns, including heatmaps, was carried out using TBtools (version 1.121) [34].

### 2.5. Validation of Gene Expression by RT-qPCR

To validate transcriptome expression profiles, a subset of ten DEGs associated with hormone signaling, Ca^2+^ transport, ROS production, and transcriptional regulation was selected for RT-qPCR analysis. Total RNA was extracted from corolla tissues using the RaPure Total RNA Plus Kit (Magen Biotech, Guangzhou, China). First-strand cDNA was synthesized with the Hifair^®^ 1st Strand cDNA Synthesis Kit (Yeasen Biotechnology, Shanghai, China) [35]. RT-qPCR was performed on a QuantStudio™ Real-Time PCR System (Applied Biosystems, Waltham, MA, USA) using the Hieff^®^ UNICON SYBR Green Master Mix (Yeasen Biotechnology, Shanghai, China). The 20 μL reaction mixture contained 10 μL of SYBR Green Master Mix, 0.4 μL of each primer (10 μM), 2 μL of cDNA template, and 7.2 μL of sterile ultrapure water. Each reaction was carried out in three biological replicates with three technical replicates. Relative expression levels were calculated using the 2^−ΔΔCt^ method [36], with actin used as the internal reference gene [37]. Primer sequences used for RT-qPCR are listed in Table S2.

### 2.6. Statistical Analysis

All statistical analyses were conducted using SPSS 25.0 (IBM, Armonk, NY, USA). Data visualization was performed in OriginPro 2024 and GraphPad Prism 8.0.1. Normality of data was tested using the Kolmogorov–Smirnov test [38], and homogeneity of variance was verified using Levene’s test [39]. One-way analysis of variance (ANOVA) followed by Duncan’s multiple range test was applied to evaluate differences among treatments [40]. Differences were considered statistically significant at *p* < 0.05.

## 3. Results

### 3.1. Overview of RNA Sequencing and Assembling

A total of 65.75 million (average of AG1–AG5), 60.50 million (average of BG1–BG5), 57.23 million (average of CG1–CG5), 55.02 million (average of DG1–DG5), and 63.21 million (average of EG1–EG5) raw reads were generated from the transcriptome libraries. After quality control, a total of 63.92 million (average of AG1–AG5), 58.75 million (average of BG1–BG5), 55.21 million (average of CG1–CG5), 53.13 million (average of DG1–DG5), and 61.43 million (average of EG1–EG5) clean reads were generated (Table 1). The quality of clean data in the *M. jalapa* corolla transcriptome was high (Table S1).

A total of 398,728 unigenes and 654,402 transcripts were assembled from transcriptome clean data (Table 2), which contained 254.41 Mb and 578.29 Mb reads, respectively. The GC content of unigenes was 38.21%, and that of transcripts was 38.52%. The largest unigene was 16,794 bp, and the smallest was 201 bp. The largest transcript was 16,794 bp, smallest was 187 bp. The average length of unigenes was 638.04 bp, but the average of transcripts was 883.69 bp. The N50 and N90 of unigenes were 883 bp and 274 bp, and the N50 and N90 of transcripts were 1509 bp and 344 bp.

### 3.2. Differentially Expressed Genes Between Five Stages Corolla of M. jalapa

**Figure 2:**
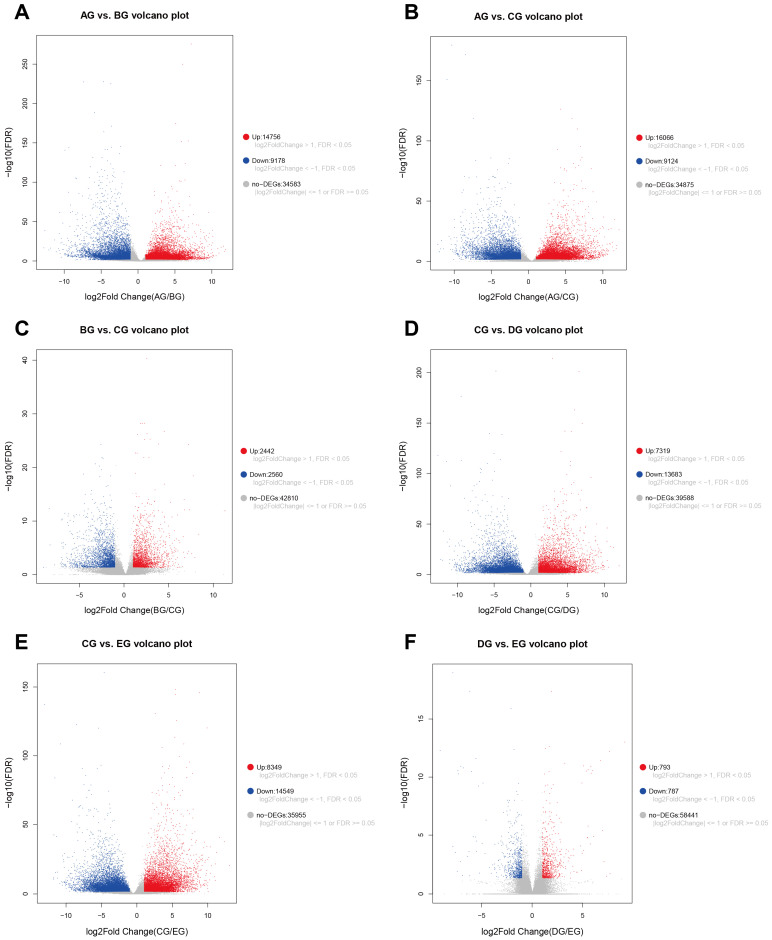
Volcano plots of differentially expressed genes (DEGs) across pairwise comparisons of five stages during *Mirabilis jalapa* corolla closure. (**A**) AG vs. BG; (**B**) AG vs. CG; (**C**) BG vs. CG; (**D**) CG vs. DG; (**E**) CG vs. EG; (**F**) DG vs. EG. Red dots represent upregulated DEGs (log_2_fold change > 1, FDR < 0.05), blue dots represent downregulated DEGs (log_2_fold change < −1, FDR < 0.05), and gray dots represent non-DEGs (|log_2_fold change| ≤ 1 or FDR ≥ 0.05).

Principal component analysis (PCA) and sample correlation heatmap both confirmed high reproducibility of biological replicates and clear transcriptional separation among different developmental stages, validating the reliability of our transcriptomic dataset (Figures S1 and S2). Differential expression analysis revealed substantial transcriptional variation among different growth groups. The results showed that 23,943 genes showed significant differential expression between AG and BG, with 14,756 upregulated and 9178 downregulated (Figure 2A). The AG versus CG comparison identified 25,190 DEGs (16,066 upregulated and 9124 downregulated) (Figure 2B). Moreover, 5002 DEGs (2442 upregulated and 2560 downregulated) were found between BG and CG (Figure 2C). In later stages, 21,002 DEGs (7319 upregulated and 13,683 downregulated) were detected between CG and DG (Figure 2D), and 22,898 DEGs (8349 upregulated and 14,549 downregulated) were observed between CG and EG (Figure 2E). Conversely, the DG–EG comparison yielded only 1580 DEGs, with 793 upregulated and 787 downregulated genes (Figure 2F).

### 3.3. GO Annotation and Enrichment

**Figure 3:**
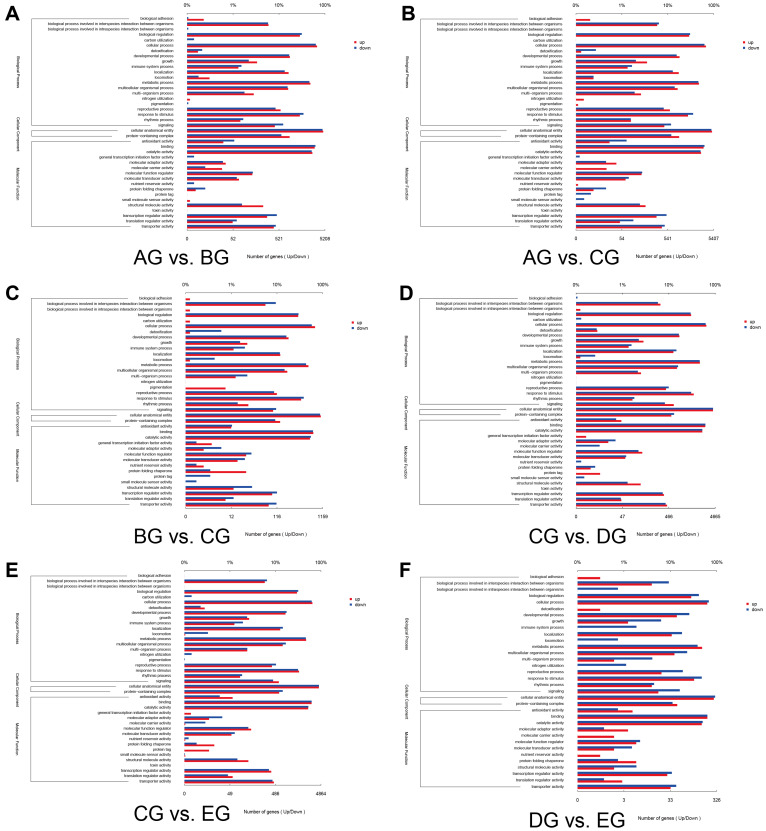
GO functional classification analysis of differentially expressed genes (DEGs) across pairwise comparisons of five stages during *Mirabilis jalapa* corolla closure. (**A**) AG vs. BG; (**B**) AG vs. CG; (**C**) BG vs. CG; (**D**) CG vs. DG; (**E**) CG vs. EG; (**F**) DG vs. EG. The x-axis indicates the number of DEGs, and the y-axis shows the GO functional categories, which are divided into three main ontologies: biological process, cellular component, and molecular function. Red bars represent upregulated DEGs, and blue bars represent downregulated DEGs.

To further elucidate the functional roles of DEGs associated with corolla movement in *M. jalapa*, Gene Ontology (GO) enrichment analysis was performed for six pairwise comparisons (AG vs. BG, AG vs. CG, BG vs. CG, CG vs. DG, CG vs. EG, and DG vs. EG) to explore the functional role of different DEGs. The top 10 significantly enriched GO categories were identified for each comparison. Most DEGs were associated with “cellular process,” “cellular anatomical entity,” and “catalytic activity,” suggesting that these biological processes and molecular functions are crucial for regulating corolla movement in *M. jalapa* (Figure 3).

### 3.4. KEGG Analysis of DEGs

**Figure 4:**
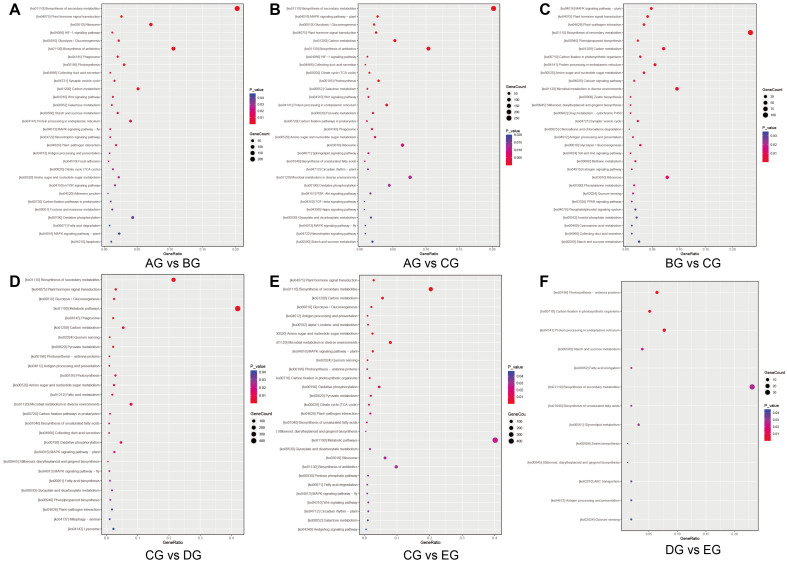
KEGG enrichment analysis of differentially expressed genes (DEGs) across pairwise comparisons of five stages during *Mirabilis jalapa* corolla closure. (**A**) AG vs. BG; (**B**) AG vs. CG; (**C**) BG vs. CG; (**D**) CG vs. DG; (**E**) CG vs. EG; (**F**) DG vs. EG. The x-axis denotes the gene ratio (the number of DEGs in a pathway divided by the total number of genes in that pathway), and the y-axis displays the top significantly enriched KEGG pathways. Dot color corresponds to the adjusted *p*-value (FDR), with redder dots indicating higher enrichment significance, while dot size represents the count of DEGs in each pathway (larger dots = more DEGs, smaller dots = fewer DEGs).

The top 30 enriched pathways indicated that DEGs across the five corolla movement stages were predominantly involved in the “mitogen-activated protein kinase (MAPK) signaling pathway–plant,” “plant hormone signal transduction,” “biosynthesis of secondary metabolites,” and “photosynthesis–antenna proteins.” Among these, the metabolic pathways showed the most significant enrichment, encompassing both “photosynthesis–antenna proteins” and “plant hormone signal transduction” (Figure 4). These pathways are closely related to the physiological regulation of plant movement.

### 3.5. Expression Changes in Plant Hormone Signalling (ko04075)

Plant hormones form a complex regulatory network that coordinates diverse physiological processes, including plant movements. Auxin (IAA), the first plant hormone discovered, is synthesized mainly in young tissues and transported to target organs via both polar and non-polar pathways, which are crucial for regulating plant movement. Key gene families in the IAA signaling pathway include *IAA*, *SAUR*, and *GH3*. In this study, there were three *IAA* genes (*TRINITY_DN3932_c1_g1*, *TRINITY_DN8494_c0_g1*, and *TRINITY_DN9752_c0_g3*) and four *SAUR* genes (*TRINITY_DN11571_c1_g1*, *TRINITY_DN30436_c1_g1*, *TRINITY_DN3652_c0_g3*, and *TRINITY_DN60805_c1_g1*) identified with high expression. Most exhibited upregulated expression under the BG and CG stages (Figure 4), suggesting a complex role for IAA in regulating *M. jalapa* corolla movement.

**Figure 5:**
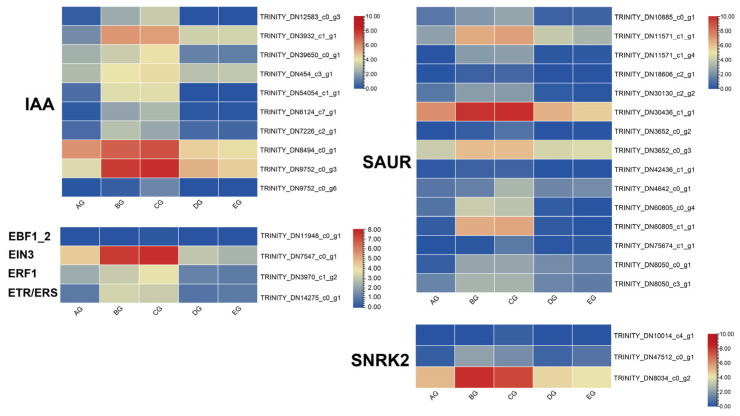
Heatmaps of expression profiles for differentially expressed genes (DEGs) involved in plant hormone signaling pathways during *Mirabilis jalapa* corolla closure.

Transcriptome analyses revealed four ethylene-related DEGs upregulated in different groups. Among them, *TRINITY_DN7547_c0_g1* showed the highest induction (Figure 4). In this study, one *SnRK2* gene (*TRINITY_DN8034_c0_g2*) was significantly enriched under the BG and CG stages (Figure 5). Collectively, these results indicate that IAA, ethylene, and ABA signaling pathways are actively involved in the corolla movement of *M. jalapa* under the BG and CG stages. However, the intricate expression patterns and potential cross-talk among these hormones require further investigation.

### 3.6. Genes Involved in Ca^2+^ Signal Pathway

**Figure 6:**
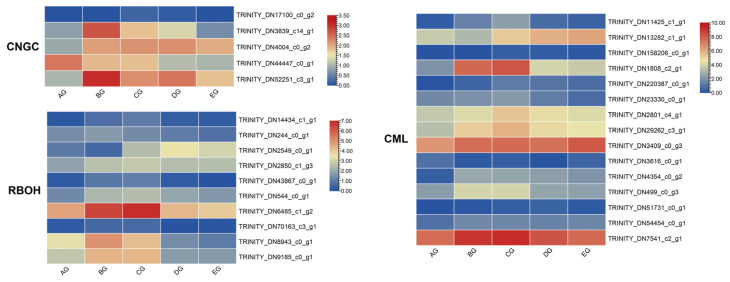
Heatmaps illustrating the expression profiles of differentially expressed genes (DEGs) involved in the Ca^2+^ signal transduction pathway during corolla closure of *Mirabilis jalapa*.

According to transcriptome data, five CNGC genes were enriched, four of which were upregulated. Among them, *TRINITY_DN52251_c3_g1* displayed the highest expression across the three stages (Figure 5). In addition, 15 calcium-binding proteins (CMLs) were enriched, with *TRINITY_DN7541_c2_g1* showing the strongest upregulation (Figure 6).

The transcriptome data revealed that ten RBOH genes were enriched, and three (*TRINITY_DN6485_c1_g2*, *TRINITY_DN8943_c0_g1*, and *TRINITY_DN9185_c0_g1*) showed upregulated expression (Figure 5). These results suggest that oxidative signaling pathways were strongly activated, producing localized ROS bursts that, together with Ca^2+^ waves, drive rapid physiological adjustments leading to movement.

### 3.7. Expression Changes in WRKY Transcription Factors

**Figure 7:**
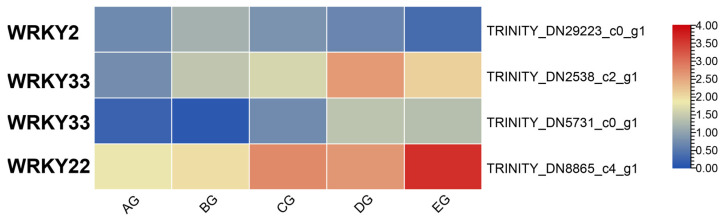
Heatmap of expression profiles for differentially expressed WRKY transcription factor genes across five stages of *Mirabilis jalapa* corolla.

According to our transcriptome results, four WRKY transcription factors (WRKY2, WRKY22, and WRKY33) were enriched. The *TRINITY_DN2538_c2_g1* (WRKY33) and *TRINITY_DN8865_c4_g1* (WRKY22) genes showed upregulation (Figure 7). Such expression changes suggest that WRKYs, activated by H_2_O_2_ and methyl viologen (MV), participate in ROS-mediated signaling cascades that promote Ca^2+^ fluxes, cytoskeletal remodeling, and cell wall modifications—processes directly linked to movement behaviors such as leaf bending, pulvinar turgor regulation, and differential growth in tropisms.

### 3.8. Verification of RNA-Seq Data by Quantitative Real-Time PCR (qRT-PCR)

**Figure 8:**
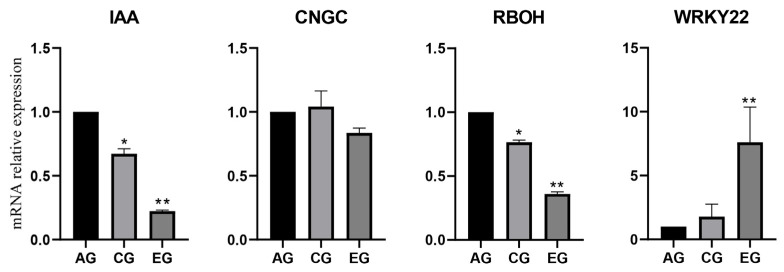
Relative mRNA expression levels of IAA, CNGC, RBOH, and WRKY22 genes detected by quantitative real-time PCR (qRT-PCR). The relative expression level of each target gene was calculated using the 2^−ΔΔCt^ method, with the AG group set as the reference (relative expression normalized to 1.0). Data are presented as mean ± standard deviation (SD) from three independent biological replicates. Asterisks indicate statistically significant differences compared with the AG group: * *p* < 0.05; ** *p* < 0.01.

To validate the reliability of our RNA-Seq transcriptome data, we selected four representative differentially expressed genes (DEGs) related to plant movement signaling (*IAA*, *CNGC*, *RBOH*, and *WRKY22*) for qRT-PCR validation. These genes are core regulators in auxin signaling, Ca^2+^ transport, reactive oxygen species (ROS) production, and stress-responsive transcription, which are key pathways mediating plant tropic and nastic movements. Relative gene expression was calculated via the 2^−ΔΔCt^ method, with the AG group set as the control. The qRT-PCR results showed that the expression trends of all target genes in the CG and EG groups were fully consistent with RNA-Seq data, with significant differential expression versus the control. These findings confirm the high reliability of our transcriptome data and support the inference that Ca^2+^-ROS-coupled signaling pathways regulate plant movement (Figure 8).

## 4. Discussion

### 4.1. GO and KEGG Enrichment of Corolla Movement

GO and KEGG enrichment analyses provided important insights into the molecular basis of corolla movement in *M. jalapa*. The enriched GO terms include “cellular process” and “catalytic activity”. This indicates that the dynamic regulation of cellular activities and molecular interactions is fundamental for the movement of the corolla [41,42]. These functions are closely associated with turgor control, cytoskeletal rearrangement, and cell wall modification, which together support the mechanical processes required for corolla opening and closing [43,44]. KEGG analysis highlighted pathways such as the MAPK signaling pathway, plant hormone signal transduction, and photosynthesis-antenna proteins. The MAPK cascade plays a central role in transmitting environmental signals to intracellular effectors and serves as a key integrator of external cues regulating corolla movements [45]. The enrichment of photosynthesis-related pathways also suggests that energy production and light perception influence rhythmic corolla activity [46]. These results collectively support the notion that corolla movement is mediated by a complex interplay of metabolic and signaling pathways that link environmental sensing with physiological and structural adjustments.

### 4.2. Plant Hormone Signalling of Corolla Movement

Plant hormones are major regulators of developmental, stress responses, and growth-related movements [47]. In *M. jalapa*, transcriptome analysis revealed that genes associated with auxin (IAA), ethylene, and abscisic acid (ABA) signaling pathways were differentially expressed across corolla developmental stages. Auxin-responsive genes (IAA, SAUR, and GH3 families) were strongly upregulated at the BG and CG stages, consistent with auxin’s role in promoting differential cell elongation and directional growth [48,49]. Such regulation likely contributes to corolla bending and expansion. In contrast, ethylene-related genes were also upregulated, reflecting ethylene’s function in fine-tuning organ movement and stress-responsive adjustments [50]. Ethylene is linked to petal expansion and senescence [51], suggesting that it may contribute to the dynamic plasticity of corolla opening. ABA-related genes, particularly SnRK2 kinases, were enriched under BG and CG stages, indicating a role for ABA in coordinating turgor regulation and stress adaptation during corolla movement [52]. The observed interaction among auxin, ethylene, and ABA implies a sophisticated hormonal network where auxin drives elongation, ethylene modulates flexibility, and ABA integrates environmental stress signals [53].

### 4.3. Ca^2+^ Signal Pathway of Corolla Movement

Calcium signaling and ROS generation constitute fundamental signaling modules in plant movement [54]. The enrichment of CNGC genes and CMLs indicates that Ca^2+^ influx and decoding are actively involved in producing oscillatory Ca^2+^ signals. This signaling regulates ion transport, cytoskeletal organization, and cell wall dynamics, all of which are required for rapid corolla adjustments [55]. ROS production, primarily mediated by RBOHs, was also enhanced, with several genes showing upregulation [56]. RBOH-generated ROS acts downstream of Ca^2+^ and, through a feedback loop, further amplifies Ca^2+^ influx to generate oscillations [57]. These Ca^2+^–ROS waves are recognized as critical regulators of turgor-driven nastic movements and tropisms [58]. In the corolla of *M. jalapa*, their activation suggests that oxidative signaling contributes directly to the execution of opening and closing movements by coordinating localized cell expansion and mechanical changes.

### 4.4. Transcription Factors of Corolla Movement

Transcription factors act as central regulators, linking environmental stimuli to gene expression programs [59]. Among the enriched transcription factor families, WRKY TFs were particularly prominent. WRKYs are established mediators of ROS–Ca^2+^ signaling and play important roles in both stress responses and movement regulation [60]. In this study, WRKY2, WRKY22, and WRKY33 showed clear upregulation, indicating that they may act as key transcriptional regulators in corolla movement. WRKY33, in particular, is known to integrate oxidative stress responses with growth regulation, suggesting a dual role in protecting cellular integrity and promoting movement [61]. Their induction by H_2_O_2_ and Methyl Viologen (MV) further highlights their role in ROS-mediated reprogramming. These findings suggest that WRKY TFs form an essential regulatory hub, coordinating Ca^2+^–ROS signaling with gene expression changes that enable corolla movement [62].

### 4.5. Expression Analysis and RT-qPCR of Key Genes

We selected four core differentially expressed genes closely associated with rhythmic corolla movement regulation, namely *IAA*, *CNGC*, *RBOH*, and *WRKY22*, for targeted expression verification. The qRT-PCR-derived expression profiles of these target genes were highly consistent with the RNA-Seq results, with a strong linear correlation between the two datasets. These selected DEGs encode key regulators of auxin signaling, Ca^2+^ transport, ROS generation, and transcriptional control, mediating the rapid cellular turgor changes and signal transduction that drive corolla movement, all of which are well-documented to be critical for plant movement-related signaling pathways [20]. The consistent expression trends further confirm the high reproducibility of our sequencing data and reinforce the conclusion that corolla movement in Mirabilis jalapa is coordinately orchestrated by the integration of phytohormone signaling, Ca^2+^–ROS regulatory modules, and transcription-factor-mediated gene expression programming [21].

## 5. Conclusions

This study provides the first comprehensive transcriptome analysis of *Mirabilis jalapa* corolla movement, revealing the molecular networks that regulate this unique floral behavior. Differentially expressed gene analysis, RT-qPCR, combined with GO and KEGG enrichment, demonstrated that auxin, ethylene, and ABA signaling, together with Ca^2+^–ROS modules, play central roles in orchestrating corolla dynamics. The enrichment of CNGCs, CMLs, and RBOHs and the strong induction of WRKY transcription factors indicate that hormone signaling, ion fluxes, and oxidative signals were tightly integrated to drive turgor regulation, cytoskeletal remodeling, and differential cell expansion. Collectively, these results establish a mechanistic framework in which plant hormones, Ca^2+^–ROS signaling, and WRKY-mediated transcriptional regulation jointly coordinate corolla movement in *M. jalapa*. This work not only advances our understanding of the molecular basis of plant movement but also provides a valuable genomic resource for future studies on floral dynamics, pollination biology, and adaptive strategies in angiosperms.

## Figures and Tables

**Table 1:** Comparison of transcriptome data statistics before and after quality control of *M. jalapa*.

Sample_ID	Raw_Total_Reads	Raw_Q20_Rate(%)	Raw_Q30_Rate(%)	Clean_Total_Reads	Clean_Q20_Rate(%)	Clean_Q30_Rate(%)
AG1	65,643,556	96.63	91.72	63,944,150	98.39	94.26
AG2	56,150,800	96.61	91.68	54,536,882	98.36	94.18
AG3	56,358,376	96.40	91.27	54,604,570	98.26	93.93
AG4	65,678,124	96.77	91.80	63,995,432	98.29	94.01
AG5	84,905,872	96.70	91.66	82,521,158	98.26	93.91
BG1	61,453,054	96.43	91.27	59,555,938	98.23	93.84
BG2	60,409,992	96.44	91.52	58,815,562	98.39	94.30
BG3	81,258,874	96.54	91.53	79,015,174	98.31	94.10
BG4	44,792,882	96.34	91.37	43,430,046	98.39	94.27
BG5	54,592,156	96.48	91.56	52,943,236	98.39	94.28
CG1	50,154,310	96.51	91.61	48,674,322	98.39	94.28
CG2	56,370,792	96.66	91.78	54,858,536	98.38	94.25
CG3	66,426,606	95.84	90.69	62,818,320	98.33	94.20
CG4	56,245,040	96.34	91.24	54,579,788	98.28	93.99
CG5	56,932,138	96.54	91.56	55,132,654	98.35	94.15
DG1	51,639,654	95.62	89.63	49,365,890	97.8	92.73
DG2	50,001,644	96.27	91.25	48,331,388	98.37	94.21
DG3	51,999,958	96.33	91.27	50,187,196	98.33	94.13
DG4	58,463,990	96.30	91.23	56,624,738	98.33	94.11
DG5	63,002,420	96.49	91.42	61,150,352	98.28	93.98
EG1	68,034,262	96.72	91.76	66,258,360	98.3	94.04
EG2	66,074,978	96.49	91.20	64,029,504	98.11	93.54
EG3	72,228,674	96.57	91.53	70,182,066	98.29	94.00
EG4	69,635,536	96.43	91.48	67,584,482	98.38	94.23
EG5	40,093,318	96.63	91.57	39,084,190	98.26	93.91

**Table 2:** Statistical table of transcriptome assembly results of *M. jalapa*.

Type	Unigene	Transcripts
Total sequence num:	398,728	654,402
Total sequence base:	254,406,292 bp	578,285,826 bp
Percent GC:	38.21%	38.52%
Largest:	16,794 bp	16,794 bp
Smallest:	201 bp	187 bp
Average:	638.04 bp	883.69 bp
N50:	883 bp	1509 bp
N90:	274 bp	344 bp

## Data Availability

All data are provided within this manuscript.

## Supplementary Materials

The following supporting information can be downloaded at https://www.mdpi.com/article/10.3390/biology15070585/s1. Figure S1: Sample-to-sample distance heatmap and correlation analysis; Figure S2: Principal component analysis (PCA) of transcriptome data; Table S1: Comparison of data statistics before and after quality control; Table S2: Primers used for qRT-PCR validation of four key differentially expressed genes.
