# Supplementary material for: Transcriptome Analysis Revealed Potential Regulatory Networks Underlying Corolla Movement in Mirabilis jalapa (Nyctaginaceae)
Source: Biology (Basel). 2026 Apr 6;15(7):585. doi: 10.3390/biology15070585 (PMC13072277; doi:10.3390/biology15070585)

Table S1 Comparison of data statistics before and after quality control.

Sample_ID	Raw_Total_Reads	Raw_Q20_Rate(%)	Raw_Q30_Rate(%)	Clean_Total_Reads	Clean_Q20_Rate(%)	Clean_Q30_Rate(%)
AG1	65,643,556	96.63	91.72	63,944,150	98.39	94.26
AG2	56,150,800	96.61	91.68	54,536,882	98.36	94.18
AG3	56,358,376	96.4	91.27	54,604,570	98.26	93.93
AG4	65,678,124	96.77	91.8	63,995,432	98.29	94.01
AG5	84,905,872	96.7	91.66	82,521,158	98.26	93.91
BG1	61,453,054	96.43	91.27	59,555,938	98.23	93.84
BG2	60,409,992	96.44	91.52	58,815,562	98.39	94.3
BG3	81,258,874	96.54	91.53	79,015,174	98.31	94.1
BG4	44,792,882	96.34	91.37	43,430,046	98.39	94.27
BG5	54,592,156	96.48	91.56	52,943,236	98.39	94.28
CG1	50,154,310	96.51	91.61	48,674,322	98.39	94.28
CG2	56,370,792	96.66	91.78	54,858,536	98.38	94.25
CG3	66,426,606	95.84	90.69	62,818,320	98.33	94.2
CG4	56,245,040	96.34	91.24	54,579,788	98.28	93.99
CG5	56,932,138	96.54	91.56	55,132,654	98.35	94.15
DG1	51,639,654	95.62	89.63	49,365,890	97.8	92.73

DG2	50,001,644	96.27	91.25	48,331,388	98.37	94.21
DG3	51,999,958	96.33	91.27	50,187,196	98.33	94.13
DG4	58,463,990	96.3	91.23	56,624,738	98.33	94.11
DG5	63,002,420	96.49	91.42	61,150,352	98.28	93.98
EG1	68,034,262	96.72	91.76	66,258,360	98.3	94.04
EG2	66,074,978	96.49	91.2	64,029,504	98.11	93.54
EG3	72,228,674	96.57	91.53	70,182,066	98.29	94
EG4	69,635,536	96.43	91.48	67,584,482	98.38	94.23
EG5	40,093,318	96.63	91.57	39,084,190	98.26	93.91

Table S2 Primers used for qRT-PCR validation of four key differentially expressed genes.

Primer name	Primer sequences (5'to3')
MjActin-F	GAAAAGCTTGCCTATGTCGC
MjActin-R	GCACCGATTGTGATGACTTG
IAA-F	GAGCACAAGTAGTCGGTTGG
IAA-R	CAGCACCATTCGTCGTCTTC
CNGC-F	TGGTTGAGTTGGTTCCTTGAC
CNGC-R	GTGGAGATTCAAACCCAGGTC
RBOH-F	AAGTGTGGTGACTGGACACA
RBOH-R	AGCACCATATGGCCCATCTAT
WRKY22-F	CGTCGATGGAATTTCCTTGGTT
WRKY22-R	AAAGTTGGTGGGCATCATCC

F

Figure S1 Sample-to-sample distance heatmap and correlation analysis. The heatmap shows the Pearson correlation coefficients between samples. The color intensity indicates the degree of correlation (red for high correlation, blue for low correlation). AG, BG, CG, DG, and EG represent five consecutive developmental stages of *Mirabilis jalapa* corolla movement.

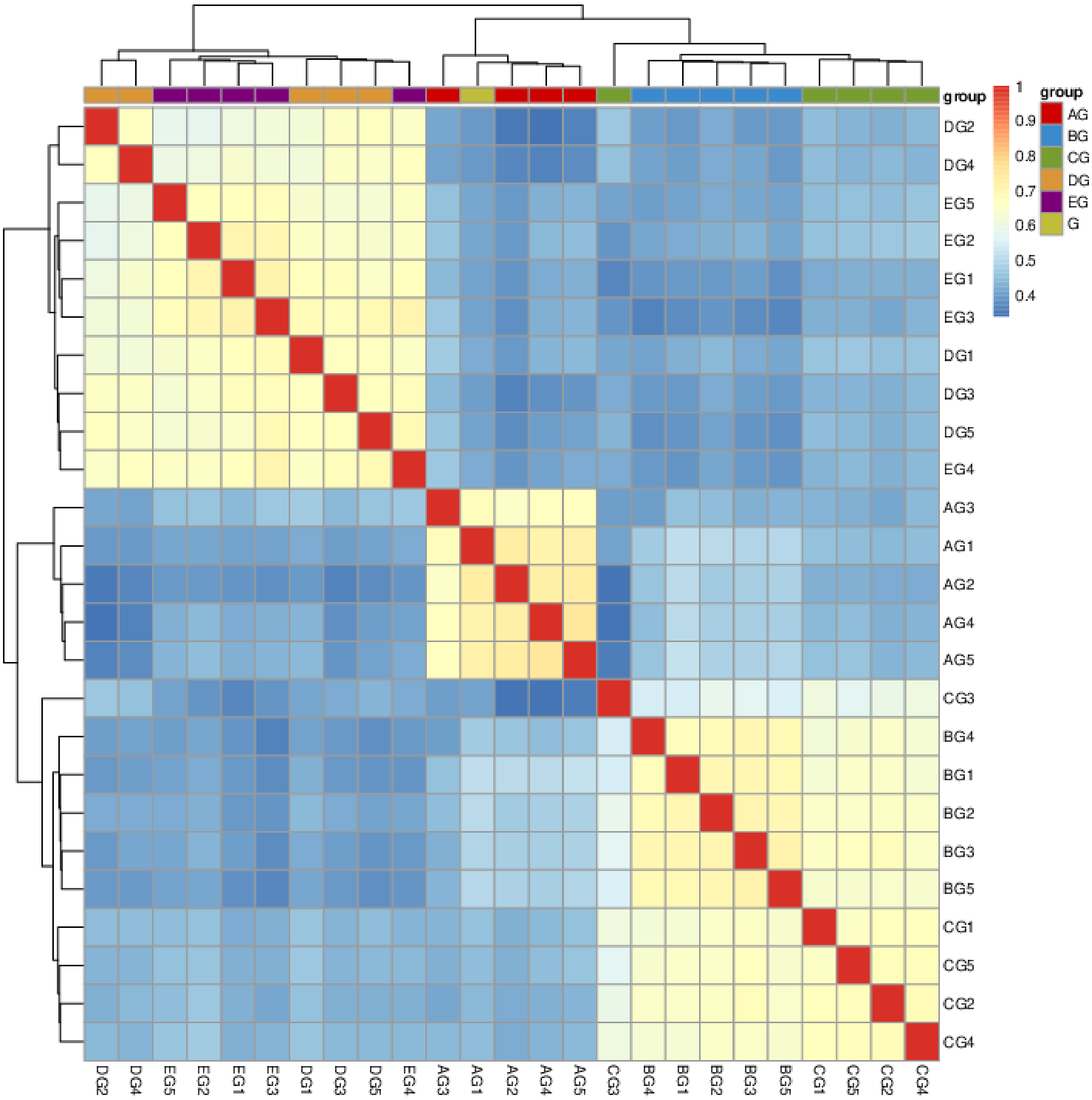

Figure S2 Principal Component Analysis (PCA) of transcriptome data. PCA was performed based on the FPKM values of all assembled unigenes. Each color represents a different developmental stage (AG: red, BG: blue, CG: orange, DG: green, EG: purple). PC1 and PC2 indicate the first and second principal components, with the percentage of variance explained shown in parentheses.

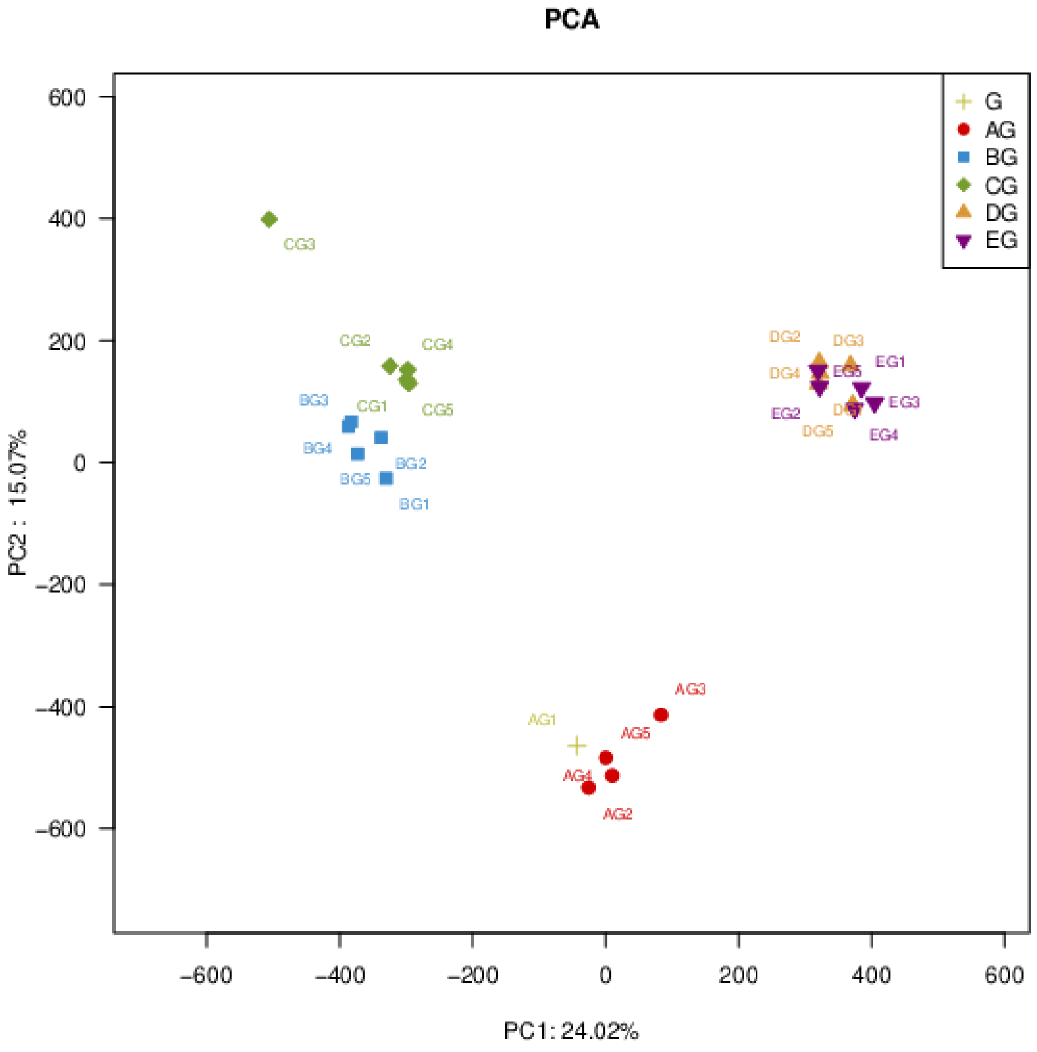

Supplement: Supplementary file 1 [file biology-15-00585-s001.zip › biology-4004520-supplementary.pdf]
